# Use of Low-Cost Magnetic Materials Containing Waste Derivatives for the (Photo)-Fenton Removal of Organic Pollutants

**DOI:** 10.3390/ma12233942

**Published:** 2019-11-28

**Authors:** Paola Calza, Jessica Di Sarro, Giuliana Magnacca, Alessandra Bianco Prevot, Enzo Laurenti

**Affiliations:** 1 Dipartimento di Chimica, Università di Torino, via P. Giuria 7, I-10125 Torino, Italygiuliana.magnacca@unito.it (G.M.); enzo.laurenti@unito.it (E.L.); 2 Nanostructured Interfaces and Surfaces (NIS) and National Interuniversity Consortium of Materials Science and Technology (INSTM) Reference Centre, Via P. Giuria 7, I-10125 Torino, Italy

**Keywords:** magnetic nanoparticles, urban waste, Fenton, pollutants, magnetite, coprecipitation synthesis

## Abstract

Hybrid magnetite/maghemite nanoparticles (MNP) coated with waste-sourced bio-based substances (BBS) were synthesized and studied for the degradation of phenol, chosen as a model pollutant, in water. A systematic study was undertaken in order to rationalize MNP–BBS behavior and optimize their performance. The effect of experimental parameters, such as light irradiation, addition of hydrogen peroxide, and the ratio between hydrogen peroxide and MNP–BBS concentrations, was studied. The generation of hydroxyl radicals was assessed, and the recovery and re-cycle of the material was investigated. Our results indicate that phenol degradation could be attained by both Fenton and photo-Fenton processes, with higher efficiency in dark condition and in the presence of a suitable amount of hydrogen peroxide. Evidence was obtained for the roles of iron ions leached from the materials as well as of organic matter released in the solution upon partial photodegradation of the organic coating. The reusability tests indicated a lower but still valid performance of the material. Optimization of the experimental conditions was performed to achieve the highest efficiency in substrate degradation, and fundamental insights into the mechanism of the MNP–BBS Fenton-like reaction were obtained.

## 1. Introduction

The United Nations General Assembly has acknowledged access to water as a fundamental human right [1]. The needs to preserve global freshwater resources and water-related ecosystems, to attain universal and equitable access to drinking water and sanitation, and to prepare for water-related disasters is unwaveringly included in the targets of several of the United Nations Sustainable Development Goals [2]. In this context, an aspect raising concern is the awareness of the presence, even in effluents from water treatment facilities, of the so-called contaminants of emerging concern (CECs). They consist of a wide range of xenobiotic chemicals (from pharmaceuticals and personal care products to persistent organic pollutants used in many industrial processes).

Many of these substances have been demonstrated to be harmful to the environment, public health, and aquatic systems [3], and therefore their complete removal through an effective tertiary water treatment is required. Among the available technologies that may be used to remove these pollutants, advanced oxidation processes (AOPs), consisting in the generation of highly reactive species which are able to mineralize most organic molecules, yielding CO_2_ and inorganic ions as final products, are considered a viable option [4]. There is, therefore, great interest in the preparation of new materials, highly performing in AOPs [5,6,7]. Among AOPs, iron-based technologies, pioneered by Fenton and photo-Fenton processes [8,9], have been demonstrated to be efficient and promising for the removal of organic recalcitrant contaminants [10,11]. They are based on the reaction between hydrogen peroxide as an oxidant and iron ions as a catalyst to produce highly reactive **·**OH radicals with an oxidation potential of 2.8 V. The need of operating at acidic pH and the occurrence of iron precipitation, which are the main drawbacks of Fenton (and related) processes, have prompted relevant research effort to optimize the operational parameters. A significant advance has been made by the use, in recent years, of many Fe(III)/Fe(II) complexes in the presence of H_2_O_2_, as homogeneous Fenton reagent. They have been indeed demonstrated to be able to efficiently remove several organic pollutants, especially highly toxic chemicals and recalcitrant compounds [12,13,14,15,16]. More recently, the attention has been focused on the possibility of using magnetic iron oxides (magnetite/maghemite nanoparticles, MNP), due to their unique physicochemical properties. The presence of Fe(II) can indeed improve **·**OH radicals production through the Fenton reaction [15]. Moreover, MNP can be easily separated from the heterogeneous system at the end of the treatment on the basis of their magnetic properties and reused for successive treatments. Recent work demonstrated the effectiveness of magnetic iron oxides in promoting the degradation of organic pollutants under Ultraviolet (UVA) irradiation (315–400 nm) [16,17]. Unfortunately, in a natural oxygen-containing atmosphere, these oxides are sensitive to oxidation, yielding non-magnetic hematite, and stabilization with an organic coating is usually carried out. In general, the addition of chelating anions or complexing polymers during magnetite formation enhances iron oxide stability, in particular avoiding oxidation [18,19,20].

Recently waste-sourced bio-based substances (BBS) have been proposed as coating materials [19,21,22,23] for the synthesis of MNP to be tested in pollutant abatement. This choice stems from the previously observed beneficial effect of BBS in photo-Fenton pollutant degradation [24,25]. Moreover, the use of residual biowaste-derived substances as a source of organic coating allows the valorization of urban and/or agricultural biowastes, thus re-entering them into the economic cycle, in line with the model of a circular economy, struggling for a zero-waste approach [26].

Preliminary encouraging results have been obtained when using hybrid BBS–magnetite/maghemite nanoparticles (MNP–BBS) for the photodegradation of caffeine [27,28,29]. Franzoso et al. [27] investigated different MNP–BBS with different BBS contents, demonstrating that the hybrid system MNP–BBS with the best (photo)Fenton-like activity in caffeine degradation was that containing 2% wt. of organic matter. For this reason, this work focuses on the same material in order to rationalize its behavior and optimize its performance through a systematic study. Phenol was chosen as the target substrate, the effect of experimental parameters was studied, and the recovery and re-cycle of the material was investigated.

For the preparation of MNP–BBS, coprecipitation synthesis was applied, adding BBS molecules to stabilize the MNP and avoid natural oxidation. Although some of the main problems relative to the establishment of the optimal experimental conditions for the preparation of magnetic materials (i.e., Fe(II)/Fe(III) ratio, pH, temperature, presence or absence of molecular oxygen) have been considered and discussed in the literature [30,31,32], some questions are still open. For instance, an excess of Fe(III), if present during MNP–BBS synthesis, can be excluded from the crystalline framework of the magnetite/maghemite phase and can form an amorphous, undefined hydroxo phase, imparting a red shadow to the typically brilliant black colour of magnetite/maghemite. This phase can hydrolyze, causing the decrease of the suspensions’ pH to about 3 from the expected pH 6. In this case, only a deep and accurate washing treatment allows the solubilization of the undesired Fe(III)-containing phase in order to obtain the plain hybrid material whose behaviours and features are described in several papers [19,27,28]. The role of Fe out of the structure as well as the release of Fe in solution and the possible occurrence of homogeneous and/or heterogeneous (photo)-Fenton processes, are discussed in the following sections, during phenol degradation.

## 2. Materials and Methods

### 2.1. Synthesis and Characterization

The synthesis of the magnetic material was performed through a coprecipitation reaction. For this, 3.7 g of FeCl_3_ and 4.2 g of FeSO_4_ × 7H_2_O were solubilized in 50 mL of deionized water and stirred heating until reaching the temperature of 90 °C. Then, 10 mL of NH_3_ 25% followed by 1 g of BBS solubilized in 50 mL of deionized water were added to the solution which turned immediately from orange to black, forming a thin precipitate. The solution was kept at 90 °C for 30 min, then the suspension was separated using a magnet, the supernatant was removed, and fresh deionized water was added to wash the resulting powder. The washing procedure was repeated three times in order to remove the unreacted reagents and neutralize the basic environment. At the end, the powder was put in a Petri dish and left in an oven (STF-N, Zetalab, Padova, Italy) at 70 °C overnight to be dried. The powder was named MNP–BBS.

The characterization of the powder was made following a well-established procedure previously reported [28]. N_2_ adsorption at 77 K was determined by an Accelerated Surface Area and Porosimetry System (ASAP2020, Micromeritics, Norcross, USA) and used to evaluate the specific surface area and porosity of the aggregated powder; X-Ray Diffraction analysis was performed by an X’Pert diffractometer (PRO MPD, Malvern PANalytical, Cambridge, UK) and used to determine the crystalline phase of the sample and confirm the presence of magnetite/maghemite (proved also by its magnetization properties); spectroscopic measurements were carried out by a Fourier-transformed infrared (FTIR) spectrophotometer (Vector 22, Bruker, Bruker, Billerica, MA, USA), on sample dispersion in KBr to evidence the vibrational feature of the hybrid material and confirm the presence of BBS. Thermogravimetric analysis was performed by a TA Instrument (TGA Q600 STD, TA Instruments, New Castle, DE, USA) in N_2_ atmosphere (flux 100 mL min^−1^, ramp 10 °C from room temperature to 800 °C) to evaluate the amount of the BBS coating the nanoparticles, without causing the oxidation of magnetite/maghemite to hematite. 

### 2.2. Material (Photo)-Activity

The (photo)catalytic ability of the synthesized material was tested at natural pH (3.5) by evaluating the disappearance of phenol (10 mg L^−1^), varying both the amount of material dispersed in water and the concentration of hydrogen peroxide, in order to achieve the best experimental conditions for abatement. Results were plotted as C/C_0_ ratio vs contact time, where C corresponds to the actual substrate concentration, and C_0_ to the initial substrate concentration.

Irradiation was performed in Pyrex glass cells kept under continuous magnetic stirring using a Philips lamp with maximum emission at 365 nm. The samples were subjected to Fenton and photo-Fenton processes; 5 mL of methanol was added to stop the reaction, and then the samples were filtered through 0.45 μm Polytetrafluoroethylene (PTFE) filters to remove the catalyst.

The production of hydroxyl radicals during the irradiation of MNP–BBS was studied by Electron Paramagnetic Resonance (EPR) in the presence of 5,5-dimethyl-1-pyrroline-N-oxide (DMPO) as a spin trap. EPR spectra were recorded at room temperature with a Bruker ESR 300E X-band spectrometer and acquired by using a previously developed method [33]: 10 μL of DMPO was added to 5 mL of a 1000 mg L^−1^ MNP–BBS suspension, and the resulting mixture was irradiated for 5 min. Immediately after the irradiation, the mixture was transferred to a quartz capillary tube, and the EPR spectrum was recorded by using the following experimental parameters: frequency = 9.78 GHz, microwave power = 5 mW, centre field = 3475 G, sweep width = 80 G, receiver gain = 1 × 10^5^, modulation amplitude = 0.41 G, conversion time = 40.96 ms, number of scans = 30.

### 2.3. Analytical Procedures

The samples are analyzed with a HPLC–UV (Merck Hitachi L-) equipped with a Rheodyne injector, two, a RPC18 Lichrochart (Merck) 12.5 × 0.8 cm column, and a UV–Vis Hitachi L-4200 detector at λ = 220 nm. Isocratic elution was performed with 80% phosphate buffer 10^−2^ M at pH 2.8 and 20% acetonitrile.

Total iron and Fe(II) were detected using a colorimetric method [34], and the analyses were performed using a UV–Vis spectrophotometer (CARY 100, Agilent, Santa Clara, USA). Fe(II) was analyzed using 4 mL of sample added with 1 mL of acetate buffer and 1 mL of phenantroline 0.1%. Total iron was determined by adding an excess of ascorbic acid to the sample and using the same procedure followed for Fe(II) analysis.

The fluorescence spectra (as excitation emission matrices, EEMs) of the filtered solutions were obtained with a Varian Cary Eclipse fluorescence spectrofluorimeter (Agilent, Santa Clara, USA), adopting a 10 nm slit width. The parameters set for conducting the analysis were: λ emission 220–600 nm; λ excitation 210–500 nm.

Total organic carbon (TOC) measurements were carried out by a TOC–VCSH Shimadzu meter (Kyoto, Japan). Calibration of the instrument was performed using standards of potassium phthalate.

## 3. Results and Discussion

### 3.1. MNP–BBS characterization

Spherical nanoparticles made of magnetite/maghemite crystals, with a statistical size distribution centered at about 20 nm of diameter, were produced (Figure S1) The organic matter surrounding the as-prepared MNP and revealed by FTIR analysis (Figure S2) was quantified in about 23% of the particle mass, as determined by TGA (see Section 3.4 for details).

The coating caused high aggregation of the dry material, presenting the N_2_ adsorption isotherm reported in Figure S3, a specific surface area of 18 m^2^ g^−1^, and an interparticle mesoporous porosity of 0.06 cm^3^ g^−1^. Some red shadows visible, in particular, at the air–particles interface, suggested the presence of a small amount of an amorphous hydroxo-Fe(III) phase in the sample, not detectable by XRD (Figure S4). The degradation tests affected the amount of organic matter coating the magnetic crystalline core (see Section 3.4) without modifying the XRD pattern (only the soluble impurities left during the synthetic pathway disappeared after the tests), whereas, as reported in [16], the aggregation of the nanoparticles decreased, decreasing the amount of organic material in the hybrid system.

The results of the characterization were in agreement with those previously reported for a similar material [27] and normally observed for magnetic nanoparticles obtained with the co-precipitation method [30,35,36].

### 3.2. Phenol Abatement in the Dark through a Fenton-Like Process

Preliminary studies showed a negligible adsorption of phenol on the MNP–BBS surface and a lower photoreactivity under UV-A light in the presence of MNP–BBS in the concentration range of 100–1000 mg L^−1^ (Figure S5). H_2_O_2_ was therefore added to the system in order to exploit MNP as a source of iron and BBS as a complexing agent to promote Fenton and photo-Fenton-like processes and improve the material performance. 

Firstly, the contribution of the sole oxidizing action of hydrogen peroxide to the abatement of phenol was tested. For this purpose, experiments were carried out in the absence of a catalyst, using a phenol solution at 10 mg L^−1^ and H_2_O_2_ at a concentration of 5 × 10^−4^ M or 1 × 10^−3^ M. In both cases, H_2_O_2_ was not able to promote the degradation of phenol (data not shown).

Subsequently, hydrogen peroxide was added to an MNP–BBS suspension, monitoring phenol degradation. In order to establish the best working conditions, experiments were carried out by varying the ratio hydrogen peroxide/MNP–BBS, keeping the phenol concentration constant.

**Figure 1:**
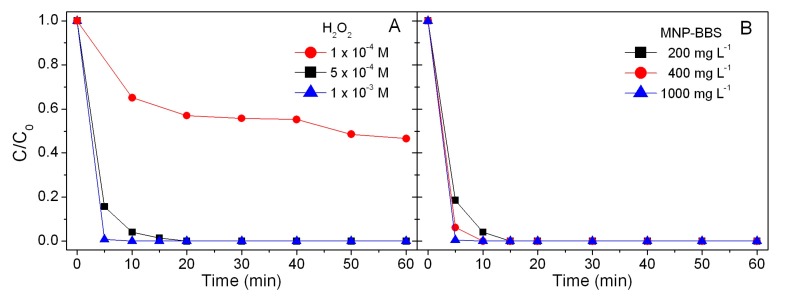
Degradation profiles for phenol (10 mg L^−1^) with (**A**) different H_2_O_2_ concentrations and magnetite/maghemite nanoparticle–bio-based substances (MNP–BBS) 200 mg L^−1^ and (**B**) H_2_O_2_ at 5 × 10^−4^ M in the presence of different MNP–BBS concentrations.

**Figure 2:**
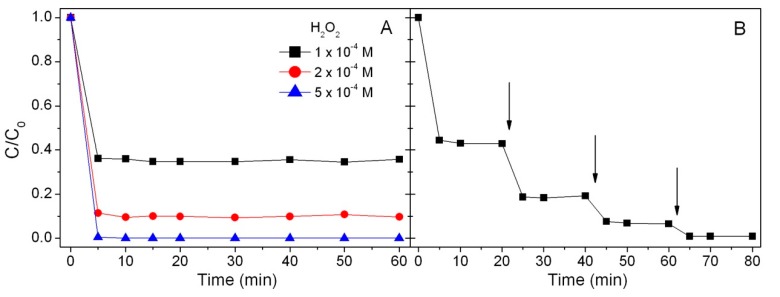
Degradation of phenol in the presence of 1000 mg L^−1^ MNP–BBS, (**A**) using different H_2_O_2_ concentrations and (**B**) following sequential addition of H_2_O_2_ 1 × 10^−4^ M (indicated by arrows).

By increasing the concentration of hydrogen peroxide, it was possible to achieve complete phenol degradation (see Figure 1A). The tests performed by using H_2_O_2_ 5 × 10^−4^ M and varying MNP–BBS concentration produced excellent results and promoted the complete degradation of phenol within 5–10 min (Figure 1B). In particular, the abatement rate increased by enhancing MNP–BBS concentration and, in the presence of MNP–BBS at 1000 mg L^−1^, the degradation was complete in only 5 min. Further investigation was performed by keeping MNP–BBS at 1000 mg L^−1^ and working with decreasing amounts of H_2_O_2_ in order to establish the specific H_2_O_2_ involvement in the reaction (see Figure 2A).

The phenol degradation profiles showed that the percentage of abatement was related to the concentration of H_2_O_2_. Phenol completely disappeared only when adding hydrogen peroxide at 5 × 10^−4^ M, while the addition of lower concentrations of H_2_O_2_ allowed only a partial degradation. These results suggest that the amount of OH· radicals produced by iron in the presence of H_2_O_2_ and typical of a Fenton reaction [27,37] is not enough to promote the complete disappearance of the substrate, thus underlining that the limiting factor in the reaction is the amount of hydrogen peroxide and not the amount of soluble iron. To confirm this hypothesis, a further test was carried out in the same conditions, proceeding with periodic additions of hydrogen peroxide (1 × 10^−4^ M) once a steady state was reached (see Figure 2B). The obtained results confirmed that the reaction stopped when the hydroxyl radicals were consumed and that the addition of fresh hydrogen peroxide allowed generating further hydroxyl radicals which could continue to attack the organic substrate.

**Figure 3:**
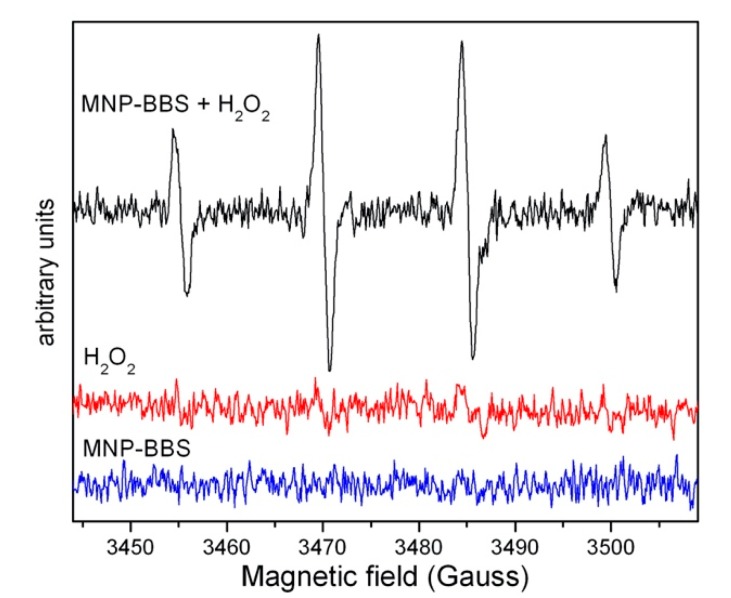
Electron paramagnetic resonance (EPR) spectra of 5-dimethyl-1-pyrroline-N-oxide (DMPO)–OH adducts generated after 5 min irradiation of MNP–BBS solutions, with and without the addition of hydrogen peroxide, and spectrum of H_2_O_2_ alone.

The formation of OH**·** radicals was assessed via EPR measurements in the presence of DMPO as a spin trap (Figure 3). In agreement with previous data, an intense typical EPR spectrum of the DMPO–OH adduct (four lines separated by 15 Gauss and with relative intensity 1:2:2:1) was clearly visible when H_2_O_2_ was added to the MNP–BBS solution, since in these conditions, iron released by MNP–BBS catalyzed the photo-Fenton reaction, leading to a high production of hydroxyl radicals. On the contrary, in the absence of the catalyst, the production of OH· radical was reduced, and moreover, in the absence of H_2_O_2_, the generation of hydroxyl radical was negligible.

### 3.3. Photo-Fenton Process: Influence of Irradiation

**Figure 4:**
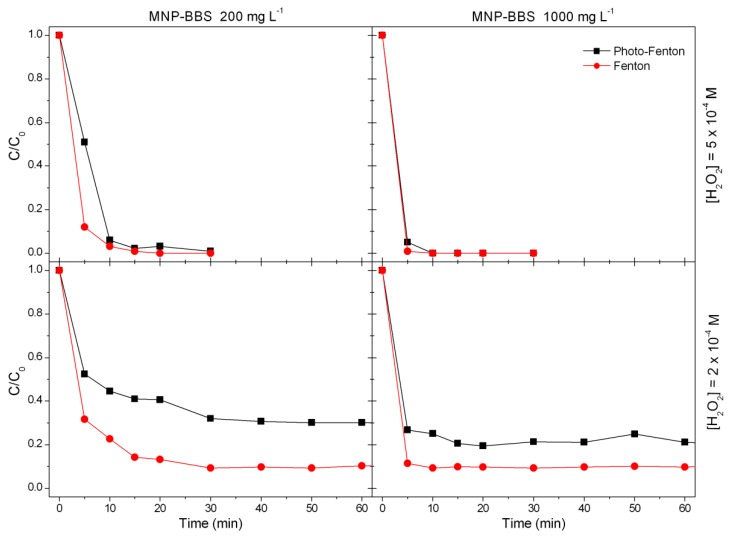
Phenol disappearance via Fenton and photo-Fenton processes with H_2_O_2_ at 5 × 10^−4^ M (top) and 2 × 10^−4^ M (bottom), as a function of MNP–BBS concentration.

Further tests were carried out to define the role of light radiation in the process. For this purpose, firstly, a solution of phenol at 10 mg L^−1^ was added with hydrogen peroxide at 5 × 10^−4^ M and was irradiated under UV-A light. The obtained results showed that phenol did not undergo significant degradation, in agreement with the literature [38]. Subsequently, phenol degradation was performed using the same experimental conditions employed in the Fenton-like experiments, but by irradiating the samples with UV-A light, in order to assess whether radiation influenced the process efficiency. A comparison of the results obtained in the two cases is shown in Figure 4.

The degradation curves for phenol were obtained as a result of Fenton and photo-Fenton processes performed while varying the concentrations of the catalyst and maintaining constant hydrogen peroxide concentration at 5 × 10^−4^ M (top) or at 2 × 10^−4^ M (bottom). Differently from what reported in the case of caffeine degradation [27], the use of light radiation seemed to inhibit the process or, at least, did not improve the performance of the material. 

### 3.4. Stability and Re-Usability: Evaluation of Released Ions/Organic Matter

**Figure 5:**
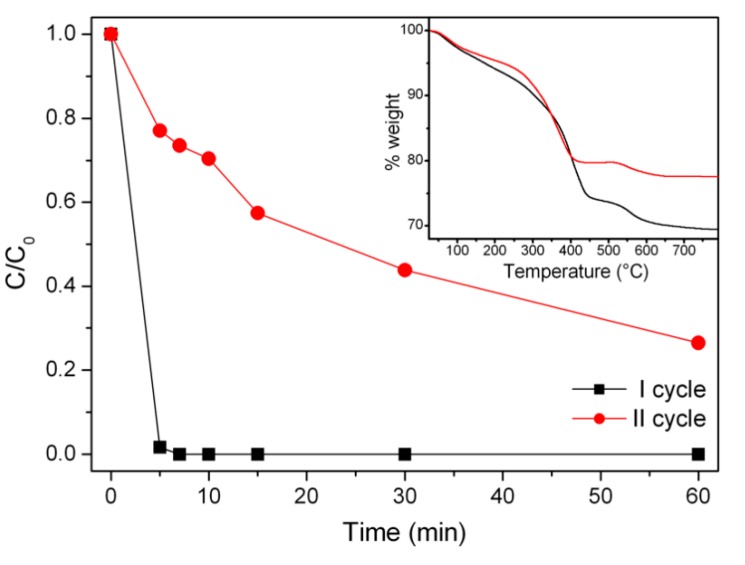
Phenol degradation via a Fenton process with MNP–BBS at 1000 mg L^−1^: I cycle (black) and II cycle (red) abatement. Inset: TGA curves of the material as-prepared (black) and after the reusability test (red).

Once the efficiency of the method was established, a test was carried out to determine if MNP–BBS recovered at the end of the Fenton-like process could be reused to carry out a new degradation cycle. For such purpose, once complete elimination of phenol was achieved, the magnetic properties of the catalyst were exploited to recover it. MNP–BBS were then washed several times with MilliQ water, dried, and re-used. The degradation curves obtained from the two subsequent cycles are compared in Figure 5.

The second cycle exhibited a lower degradation efficiency: MNP–BBS were able to promote a partial elimination of phenol, with 70% of abatement after one hour.

In order to assess whether the process induced changes on the organic phase used to coat and stabilize the magnetite nanoparticles, TGA analysis on the material after one working cycle and recovery was performed. The percentage of organic content, obtained from the weight loss in the range of 250–700 °C, was different in the two cases, and the difference corresponded to 10%. This change suggests that the reactive species generated during the Fenton process could also act on the organic coating of the nanoparticles inducing its partial decomposition. However, this loss could not be directly correlated with the decrease of efficiency found in the second cycle, and therefore it was necessary to deepen the study of this material to understand the role of the organic matter. This efficiency loss could be caused by the organic matter and the iron oxide, as both participated in the overall process. Therefore, these two actors were separately inspected and are described in the following section.

### 3.5. Release of Organic Matter

**Figure 6:**
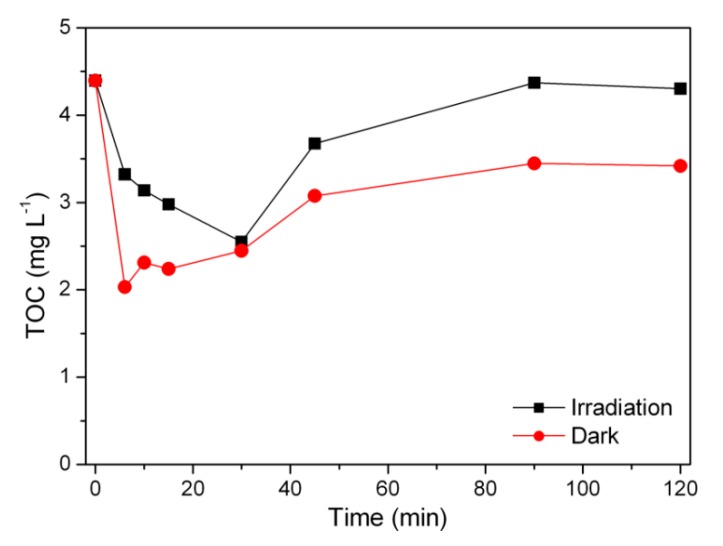
Total organic carbon (TOC) released by the catalyst in the presence of 5 × 10^−4^ M H_2_O_2._

The loss of organic matter was investigated by performing both TOC measurements (to investigate the amount of organics release) and fluorescence spectra (to define the nature of the organics release). TOC measured at time zero allowed the assessment of the amount of organic carbon released by the material, which corresponded to about 4.5 mg L^−1^. The TOC evolution profile over time was then followed after the addition of H_2_O_2_ at 5 × 10^−4^ M both in the dark and under UV-A irradiation (see Figure 6).

The addition of hydrogen peroxide caused the partial degradation of the organic fraction without involving its complete mineralization. At longer irradiation times, the material continued to release organic carbon in the solution, suggesting that this phenomenon could also cause the release in the solution of iron species derived from the magnetic iron-containing core of the nanoparticles. Under irradiation, the released TOC was higher than in the tests conducted in the dark, implying that light could act on the material degradation as well.

**Figure 7:**
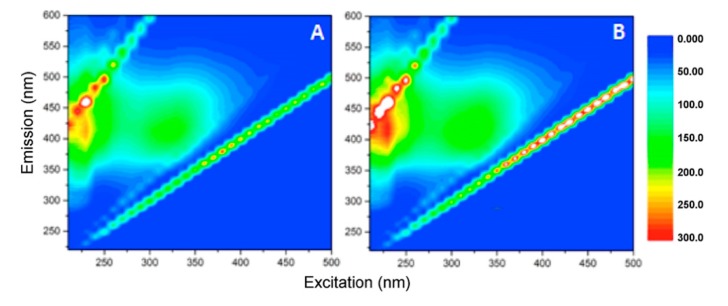
Excitation Emission fluorescence matrices (EEMs) acquired using filtered samples at time zero (**A**) and after 2 h of UV-A irradiation (**B**).

According to the literature [39], the organic substances used to coat magnetite nanoparticles show structural similarities with humic substances and, therefore, fluorescence matrix spectroscopy can be exploited to study the properties and characteristics of samples containing humic-like substances [40,41,42]. Therefore, the EEMs shown in Figure 7 were acquired using filtered samples. 

In the starting material, a very intense and broad emission peak centered at the excitation/emission coordinates 210/420 nm was evident (Figure 7A). Referring to the literature [42], this signal is attributable to the presence of humic-like components. A second but less intense fluorescence peak was observed at Ex/Em 330/410 nm, which could be attributed to humic-like and fulvic-like components, too. After 2 h of UV-A irradiation, the same pattern of peaks was still present (Figure 7B), permitting to establish that the organic matter was not photolyzed.

**Figure 8:**
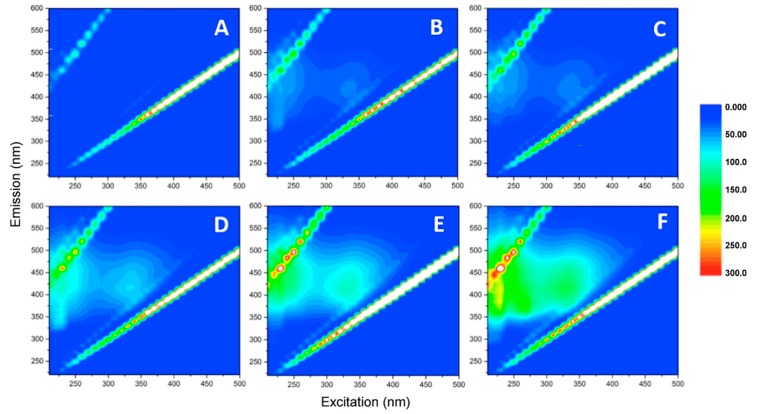
EEMs acquired after the addition of H_2_0_2_ 5 × 10^−4^ M under UV-A irradiation. Samples were analyzed after 5 min (**A**), 10 min (**B**), 15 min (**C**), 30 min (**D**), 60 min (**E**), and 120 min (**F**) of irradiation.

The samples were then supplemented with hydrogen peroxide and subjected to irradiation with a UV-A lamp for different times (Figure 8). Within few minutes, quenching of the fluorescence, with the disappearance of all signals attributable to humic-like substances, was visible. These results suggest that the action of hydrogen peroxide and light could affect the properties of some of the components of the material, destroying those responsible for fluorescence emission. These results agree with those obtained by the TOC measures, which showed a TOC decrease within 15 min after the addition of the hydrogen peroxide. New signals appeared in samples subjected to longer irradiation times (Figure 8E,F), even if with lower intensities than those observed at time zero; this implies that a prolonged irradiation could lead to the release of new fluorescent species, responsible for the increased TOC content described above (see Figure 6).

**Figure 9:**
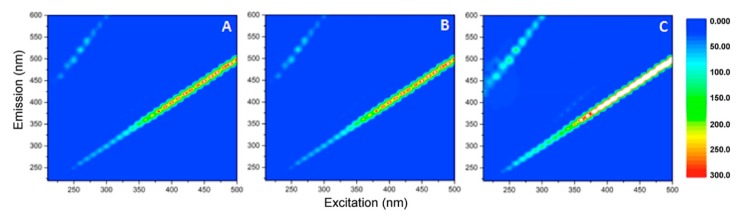
EEMs acquired for filtered samples following the addition of H_2_0_2_ at 5 × 10^−4^ M. The samples were measured after 5 min (**A**), 10 min (**B**), 15 min (**C**) of H_2_0_2_ addition at 5 × 10^−4^ M.

EEMs were also acquired in the dark and were plotted in Figure 9. These images show the disappearance of the characteristic signals after few minutes from H_2_O_2_ addition, suggesting that hydrogen peroxide acted on the organic component covering the magnetic nanoparticles, modifying the nanoparticles’ structure and/or properties also in the dark.

### 3.6. Release of Soluble Iron

Iron oxide affected the process acting as a source of soluble iron, and therefore, the release of Fe(II) and Fe(III) in solution occurring during the Fenton and photo-Fenton processes was determined by using the classic *o*-phenanthroline method [34] in the optimized experimental conditions, i.e., in the presence of 1000 mg L^−1^ of MNP–BBS and H_2_O_2_ at 5 × 10^−4^ M. In both cases, the total amount of released iron was 20 mg L^−1^, while the amount of measured Fe(II) was around 15–17 mg L^−1^; Fe(II) concentration was higher than that reported in the literature [27], probably because the working pH value of 3.5 favored the dissolution of iron cations [37].

**Figure 10:**
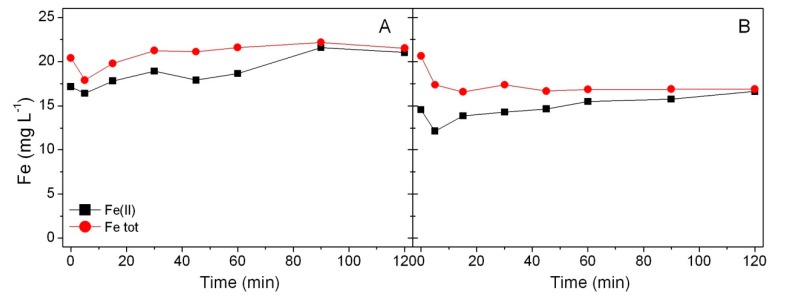
Fe(II) and total iron released during the Fenton process (**A**) and the photo-Fenton process; (**B**) in all cases, the experimental conditions were: 1000 mg L^−1^ of catalyst, 5 × 10^−4^ M of H_2_O_2._

Some interesting suggestions about the reaction mechanism can be made considering the concentration of Fe(II) in Figure 10. The highest consumption of Fe(II) was recorded within 5 min, i.e., in the period of time corresponding to the highest reaction rate observed in Fenton-like and photo-Fenton-like conditions. 

It is worth noting that a partial consumption of Fe(II) occurred in the first few minutes of the Fenton-like process, in accordance with reaction (1):

Fe^II^ + H_2_O_2_ → Fe^III^ + OH^−^ + HO^•^*k* = 40 − 80 L⋅mol^−1^s^−1^ (1)

At the beginning of the process, in fact, Fe(II) was the predominant species, and its relative concentration increased during the reaction both in Fenton-like and photo-Fenton-like conditions. At the same time, Fe(II) ions were released from iron oxide (losing its protective organic coating during (photo)Fenton-like process), as their concentration in the solution remained almost constant, whereas photoreduction of Fe(III) or complexation of Fe(III) by BBS explains the relative concentration decrease of Fe(III) in solution reported in Figure 10, which shows that after 2 h of treatment, the total iron amount corresponded to Fe(II).

The iron species revealed in the above-described measurements could derive from a slight dissolution of the magnetite/maghemite nanoparticles but also from iron out of the structure, which is easily dissolved during experiments. The contribution of the nanoparticles should remain almost constant along several reaction cycles, whereas the contribution of amorphous iron hydroxide should stop once the dissolved compound has been removed from the reaction medium. Therefore, once out-of-the-structure Fe cations are consumed (during the first reaction cycle) the reaction continues only because of the solubilization of Fe(II) from the magnetic phase caused by BBS consumption, and the reactivity slows down.

## 4. Conclusions

Phenol degradation was studied using MNP–BBS, a low-cost material easy recoverable from aqueous media thanks to its magnetic features. BBS, used as a protective organic layer against magnetite/maghemite oxidation, present the advantage of complexing iron species released from the magnetic core of the material, preventing their precipitation and increasing their availability during the catalyst activity. All the trials carried out indicated that the material is a suitable system to achieve the degradation of organic substrates in Fenton-like condition, simply adding small amounts of H_2_O_2_ to produce hydroxyl radicals. The reusability test indicated a good performance of the material, sustained by the continuous release of iron species from the MNP.

Finally, MNP–BBS is stable under UV-A radiation, but H_2_O_2_ addition induced a partial degradation of the material, with the release of organic species.

## Supplementary Materials

The following are available online at https://www.mdpi.com/1996-1944/12/23/3942/s1, Figure S1: TEM image of nanoparticles, Figure S2: IR spectrum of nanoparticles dispersed in KBr, Figure S3: Adsorption isotherm of 77 K N_2_, Figure S4: XRD diffractogram reporting the principal signal of Fe_3_O_4_ and impurities of NH_4_NO_3_ derived from the synthesis procedure, Figure S5: Degradation profiles of phenol (10 mg L^−1^) in the presence of different MNP–BBS concentrations.
