# Supplementary material for: Use of Low-Cost Magnetic Materials Containing Waste Derivatives for the (Photo)-Fenton Removal of Organic Pollutants
Source: Materials (Basel). 2019 Nov 28;12(23):3942. doi: 10.3390/ma12233942 (PMC6926715; doi:10.3390/ma12233942)
Supplement: Supplementary file 1 [file materials-12-03942-s001.pdf]

materials

Supplementary materials

Use of Low-Cost Magnetic Materials Containing Waste Derivatives for the (Photo)-Fenton Removal of Organic Pollutants

Paola Calza [1,*], Jessica Di Sarro [1], Giuliana Magnacca [1,2], Alessandra Bianco Prevot [1,*] and Enzo Laurenti [1]

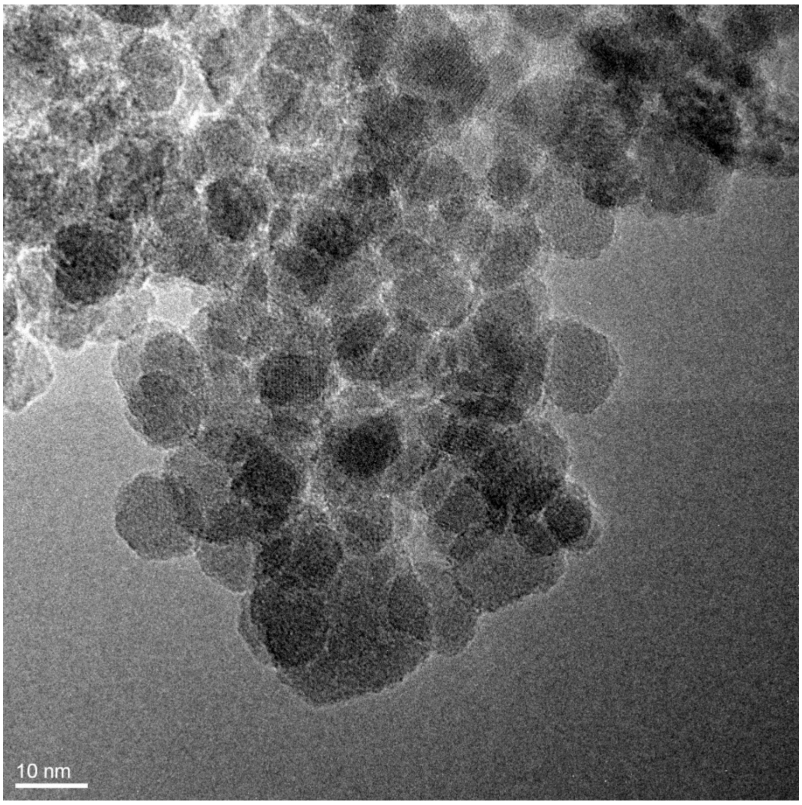

Figure S1. TEM image of nanoparticles.

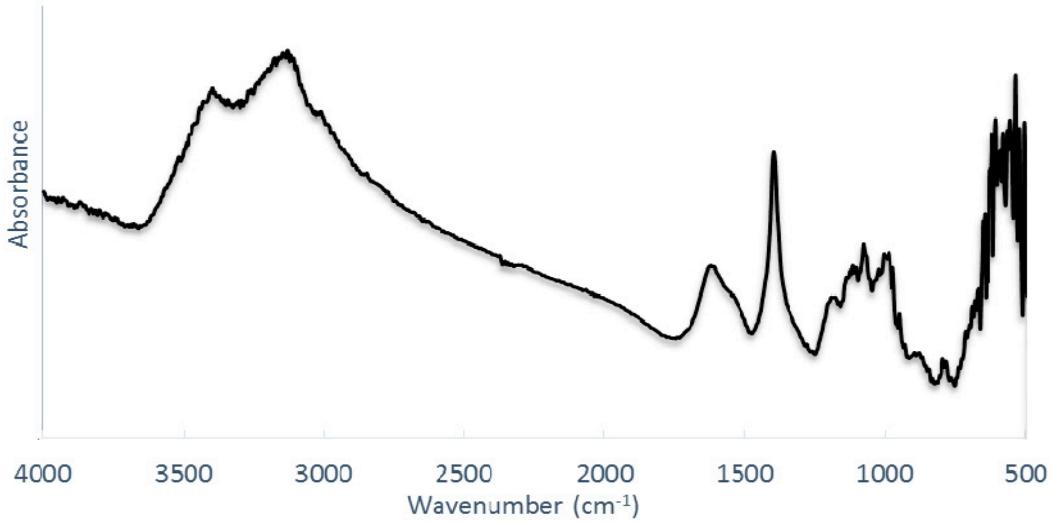

Figure S2. IR spectrum of nanoparticles dispersed in KBr.

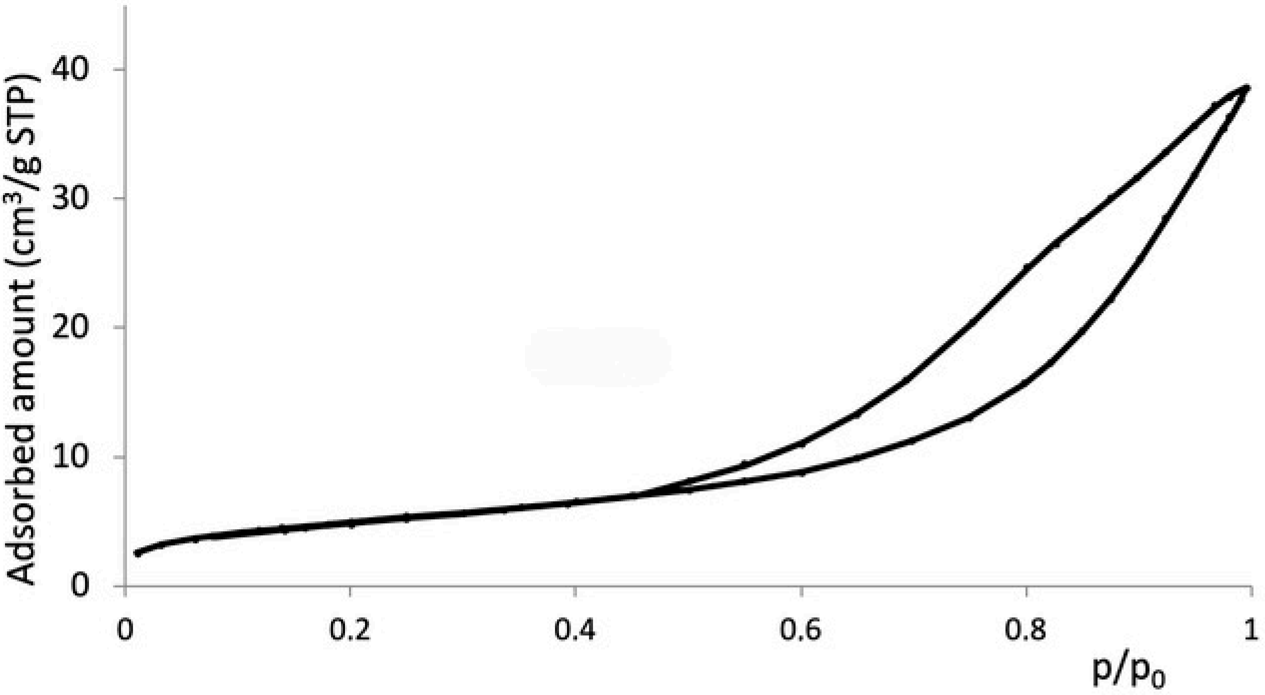

Figure S3. Adsorption isotherm of 77 K N_2.

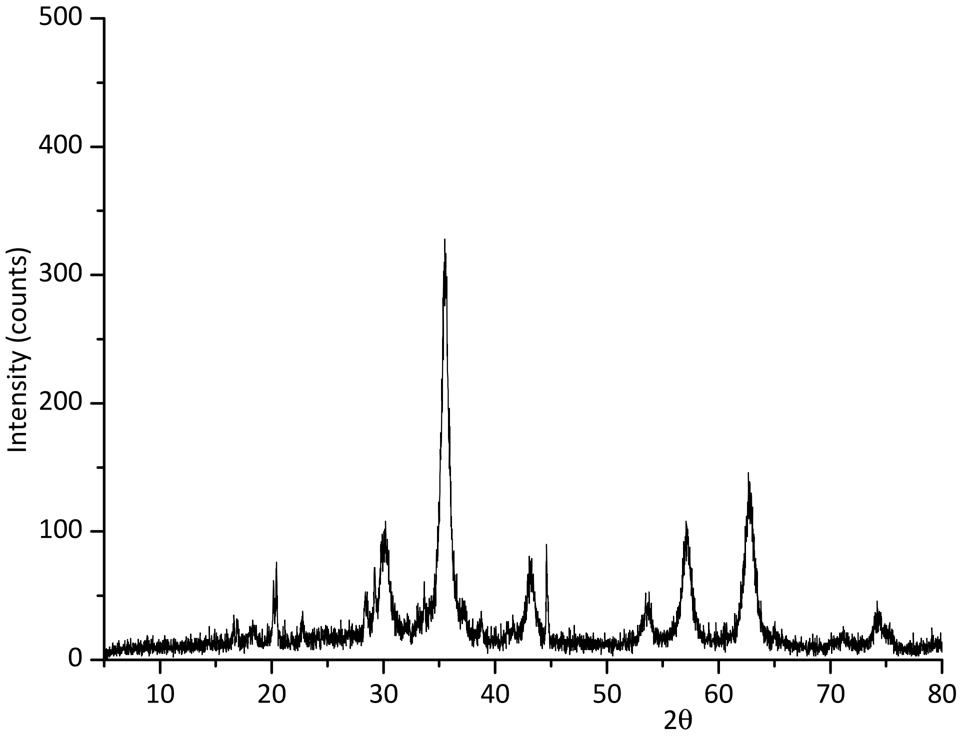

Figure S4. XRD diffractogram reporting the principal signal of Fe_3O_4 and impurities of NH_4NO_3 derived from the synthesis procedure.

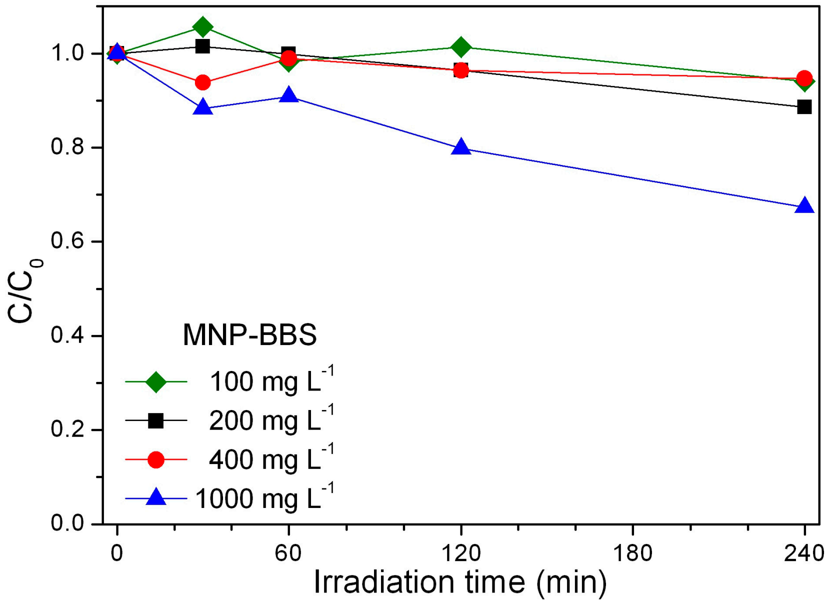

Figure S5. Degradation profiles of phenol (10 mg L^{-1}) in the presence of different MNP–BBS concentrations.

© 2019 by the authors. Submitted for possible open access publication under the terms and conditions of the Creative Commons Attribution (CC BY) license (http://creativecommons.org/licenses/by/4.0/).
